# Expression of G2019S LRRK2 in Rat Primary Astrocytes Mediates Neurotoxicity and Alters the Dopamine Synthesis Pathway in N27 Cells via Astrocytic Proinflammatory Cytokines and Neurotrophic Factors

**DOI:** 10.3390/cimb46050263

**Published:** 2024-05-06

**Authors:** Dong Hwan Ho, Hyejung Kim, Daleum Nam, Mi Kyoung Seo, Sung Woo Park, Ilhong Son

**Affiliations:** 1 InAm Neuroscience Research Center, Sanbon Medical Center, College of Medicine, Wonkwang University, 321, Sanbon-ro, Gunpo-si 15865, Republic of Korea; ceci776@naver.com (H.K.); ekfma304@naver.com (D.N.); 2 Paik Institute for Clinical Research, Inje University, Busan-si 47392, Republic of Korea; first1011486@hanmail.net (M.K.S.); neuro109@hanmail.net (S.W.P.); 3 Department of Convergence Biomedical Science, Inje University College of Medicine, Busan-si 47392, Republic of Korea; 4 Department of Neurology, Sanbon Medical Center, College of Medicine, Wonkwang University, 321, Sanbon-ro, Gunpo-si 15865, Republic of Korea

**Keywords:** Parkinson’s disease, leucine-rich repeat kinase 2 (LRRK2), astrocyte, neurotrophic factor, neuroinflammation, dopamine synthesis pathway

## Abstract

Astrocytes in the brain contribute to various essential functions, including maintenance of the neuronal framework, survival, communication, metabolic processes, and neurotransmitter levels. Leucine-rich repeat kinase 2 (LRRK2) is associated with the pathogenesis of Parkinson’s disease (PD). LRRK2 is expressed in neurons, microglia, and astrocytes and plays diverse roles in these cell types. We aimed to determine the effects of mutant human G2019S-LRRK2 (GS-hLRRK2) in rat primary astrocytes (rASTROs). Transfection with GS-hLRRK2 significantly decreased cell viability compared to transfection with the vector and wild-type human LRRK2 (WT-hLRRK2). GS-hLRRK2 expression significantly reduced the levels of nerve growth factor and increased the levels of proinflammatory cytokines (interleukin-1β and tumor necrosis factor α) compared to the vector and WT-hLRRK2 expression. Furthermore, GS-hLRRK2 expression in rASTROs promoted astrogliosis, which was characterized by increased expression of glial fibrillary acidic protein and vimentin. Treatment with the conditioned medium of G2019S LRRK2-expressing rASTROs decreased N27 cell viability compared to treatment with that of WT-hLRRK2-expressing rASTROs. Consequently, the regulation of the dopamine synthesis pathway was affected in N27 cells, thereby leading to altered levels of tyrosine hydroxylase, dopamine transporter, Nurr1, and dopamine release. Overall, the G2019S LRRK2 mutation disrupted astrocyte function, thereby aggravating PD progression.

## 1. Introduction

Astrocytes are important brain components and form a structural framework for neurons [1], maintain the blood–brain barrier [2], enhance neuronal communication [3], regulate metabolic processes [4], repair injury [5], and release neurotransmitters [6,7]. The multifaceted roles of astrocytes significantly contribute to the brain’s overall health and function, and astrocytic neurotrophic factors and inflammatory cytokines are critical for maintaining neuronal health [8,9].

The upregulation of leucine-rich repeat kinase 2 (LRRK2) activity is closely associated with the pathogenesis of Parkinson’s disease (PD) [10]. LRRK2 is expressed in the brain, particularly in neurons, microglia, and astrocytes. The roles of LRRK2 in these cells have been reported previously [11,12,13]. Notably, LRRK2 is associated with neuroinflammation, a crucial factor for PD progression [14]. Neuroinflammation in the brain is primarily mediated by microglia and astrocytes [15]. LRRK2 regulates inflammatory responses, and its kinase activity plays a key role in neuroinflammation [16]. The G2019S LRRK2 mutant, characterized by upregulated kinase activity, promotes neuroinflammation in the microglia [17]. However, the role of G2019S LRRK2 in astrocytes remains unclear. 

This study aimed to determine the effect of the G2019S LRRK2 mutant in rat primary astrocytes on astrogliosis, the expression of inflammatory cytokines and neurotrophic factors, and dopaminergic neuronal maintenance.

## 2. Methods and Materials

### 2.1. Rat Primary Astrocyte Isolation, Culture, and Transfection

Primary microglia and astrocytes were obtained from the cortex of dissected fetal rat brains (embryonic day 17 [E17]). The tissues were incubated with trypsin-EDTA (0.25%), phenol red (25200056; Gibco, Carlsbad, CA, USA), and bovine pancreas deoxyribonuclease I (DN-25-100MG; Sigma-Aldrich, St. Louis, MO, USA) for 30 min; dissociated via pipetting; and centrifuged at 200× *g* for 10 min at room temperature (RT) to remove cell debris and myelin. The resulting cells were filtered through a nylon mesh and seeded in a T75 flask (11090; SPL, Pocheon-si, Republic of Korea). The cells were maintained in DMEM/F12 (LM002-04; Welgene, Gyeongsan-si, Republic of Korea) supplemented with 10% fetal bovine serum (FBS, BFS-1000; T&I, Chuncheon-si, Republic of Korea) and 1× antibiotic–antimycotic solution (15240096, Gibco). After 1 week, the cells were detached using TrypLE™ Express Enzyme (1×), no phenol red (12604013; Gibco), and reseeded in T75 flasks for further culture. After 2 weeks, microglia cells were detached via rough tapping and stored as stock. Astrocytes were then detached using TrypLE™ Express Enzyme (1×), with no phenol red. Rat primary astrocytes (rASTROs) were seeded (5 × 10^5^) in 12-well plates (30012, SPL). For efficient transfection of rASTROs, the cells were pretreated with NATE™ (InvivoGen, San Diego, CA, USA) for 30 min, following the manufacturer’s instructions. rASTROs were then transfected with 1 μg pcDNA 3.1 (vector) carrying LRRK2 DNA, wild-type human LRRK2 (WT-hLRRK2), or mutated human G2019S LRRK2 (GS-hLRRK2) using LipoD293. The culture medium was changed on day 1 of the 5-day transfection procedure. Cell lysates were harvested using Laemmli buffer (4×, L1100-001; GenDEPOT, Katy, TX, USA) diluted with sterile distilled water. The culture medium was collected after 5 days of cell culture to serve as the conditioned medium (CM) of transfected rASTROs. The medium was centrifuged at 2100 rpm for 10 min at 4 °C, and the resulting supernatant was collected and stored at −70 °C for several days. 

### 2.2. Cell Viability Assay

To determine the viability of cells transfected with LRRK2, rASTROs were seeded in a 96-well plate and pretreated with NATE. rASTROs were then transfected with 1 μg vector, WT-hLRRK2, or GS-hLRRK2 using LipoD293. All samples were analyzed simultaneously after the 5-day transfection process; day 1 was excluded as the culture medium containing transfection reagents was replaced on this day. Each sample was treated with CCK-8 reagent (CK04, Dojindo, Kumamoto, Japan) at 1/10 of culture media volume, and the absorbance was measured at 450 nm after 2 h. To evaluate cell viability, a non-transduced sample was employed as a control for 100% viability. A control for 0% viability, in which all cells were killed by adding 10% Triton X-100 to a final concentration of 1% in culture media, was also used.

### 2.3. Isolation of mRNA and cDNA Synthesis

mRNA isolation and cDNA synthesis were performed as previously described [18].

### 2.4. Reverse Transcription PCR (RT-PCR)

RT-PCR was performed using 1 μL of synthesized cDNA, 2.5 μL 10× Ex Taq buffer, 2 μL dNTP (2.5 mM), 0.125 μL Ex Taq (RR001B; TaKaRa Bio Inc., Shiga, Japan), and 1 μL of primer. The total volume was adjusted to 25 μL using sterile RNase-free water. The PCR products were analyzed using agarose gel electrophoresis. 

### 2.5. Quantitative PCR (qPCR)

qPCR was performed using TOPreal™ SYBR Green qPCR PreMIX (RT500S; Enzynomics, Daejeon, Republic of Korea). The primers used in the experiments are listed in Table 1. The analysis was conducted using a magnetic induction cycler (Bio Molecular Systems, Upper Coomera QLD, Australia). The mRNA levels of the genes of interest were calculated using the 2^−∆∆Ct^ method.

### 2.6. Enzyme-linked Immunosorbent Assay (ELISA)

The culture media were collected from the transfected rASTROs and centrifuged at 4000 rpm for 10 min at 4 °C. The levels of cytokines, neurotrophic factors, and dopamine in the supernatants were then determined using ELISA. The following commercially available ELISA kits were employed: Rat brain-derived neurotrophic factor (BDNF) ELISA Kit PicoKine (EK0308, Boster Biological Technology, Pleasanton, CA, USA), Rat glial cell line-derived neurotrophic factor (GDNF) ELISA Kit PicoKine (EK0363, Boster Biological Technology), Rat Nerve Growth Factor (NGF) ELISA Kit (MBS261790. MyBioSource, Inc., San Diego, CA, USA), Rat tumor necrosis factor-alpha (TNFα) DuoSet ELISA (DY5100-05; R&D System, Minneapolis, MN, USA), Rat Interleukin 1β (IL-1β) ELISA Kit (CSB-E08055r; CUSABIO, Houston, TX, USA), and Dopamine ELISA kit (KA3838, Abnova, Taipei City, Taiwan). All analyses were conducted according to the manufacturers’ instructions. The concentration of each protein was estimated using the standards provided in the kit.

### 2.7. Western Blot Analysis

Lysed samples were sonicated for 20 s (amplification frequency, 10%) using an ultrasonic processor (VCX 130; Sonics & Materials, Inc., Newtown, CT, USA) and boiled at 95 °C for 5 min. Sample aliquots of 15 μL were then loaded onto a 4–20% 15-well MINI-PROTEAN^®^ TGX Precast Protein Gel (4561096; Bio-Rad, Hercules, CA, USA). Sodium dodecyl sulfate–polyacrylamide gel electrophoresis was performed at 100 V for 100 min. The resolved proteins were then electro-transferred onto a nitrocellulose membrane (10600004; Cytiva, Marlborough, MA, USA) at 300 mA for 80 min. The membranes were then immersed in 5% skim milk in Tris-buffered saline containing 0.1% Tween-20 (TBST) for 30 min at RT, incubated overnight with the primary antibodies (Table 1) and 1% bovine serum albumin in TBST, and washed thrice with TBST for 5 min at RT using a shaker. Finally, the membranes were incubated with horseradish peroxidase (HRP)-conjugated secondary antibodies to detect the protein bands. Luminata Crescendo western HRP substrate (WBLUR0500; Merck & Co., Inc., Kenilworth, NJ, USA) was used to develop immunoreactive signals on the nitrocellulose membranes. Images of the protein bands were obtained using a MicroChemi 4.2 camera (Shimadzu, Kyoto, Japan). Equal sample loading was confirmed via Coomassie blue staining. The antibodies used are listed in Table 2.

### 2.8. N27 Cell Culture and Treatment with the CM of rASTROs

N27 rat dopaminergic neuronal cells were cultured in RPMI 1640 (LM011-06, Welgene) supplemented with 10% EquaFETAL (EF-0500-A, Atlas Biologicals, Fort Collins, CO, USA) and 1× antibiotic–antimycotic solution at 37 °C in a 5% CO_2_ incubator. The N27 cells were seeded in 24-well plates (30024; SPL) and treated with the CM of rASTROs for 48 h. Cell viability was determined using the CCK-8 assay for 3 h, following the manufacturer’s protocol. The optical density at 450 nm was measured using a microplate reader. Subsequently, the cells were harvested using sample buffer. Live N27 cells were incubated with 1 μM of H2DCFDA (D399, Invitrogen, Carlsbad, CA, USA) and μM SYTO59 red fluorescent nucleic acid stain (S11341, Invitrogen) for 2 h to determine the levels of reactive oxygen species (ROS). The intensities of each fluorescence were measured at excitation/emission at 504/529 and 622/645 nm for H2DCFDA and SYTO 59 red fluorescent nucleic acid stain, respectively, using an EnVision^®^ Multilabel Plate Reader (Perkin Elmer, Waltham, MA, USA). The media were collected to measure dopamine levels.

### 2.9. Statistical Analysis

The Western blot bands were analyzed using Multi Gauge V 3.0 software (Fujifilm, Tokyo, Japan). All statistical analyses were performed using Prism 8 software (GraphPad Software, La Jolla, CA, USA). The parameters used for the statistical analyses are described in the figure legends.

## 3. Results

### 3.1. G2019S LRRK2 Expression-derived Cytotoxicity in rASTROs

**Figure 1:**
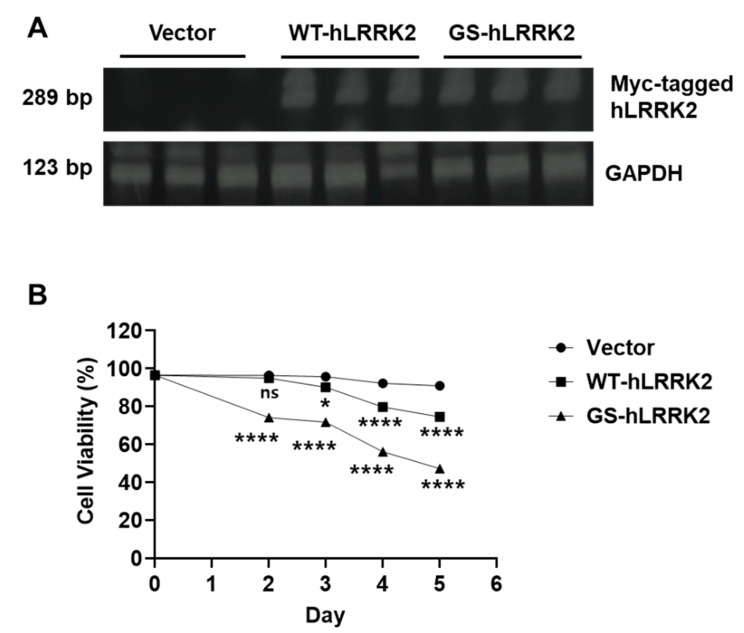
Cytotoxicity due to ectopic expression of leucine-rich repeat kinase 2 (LRRK2) in rat primary astrocytes (rASTROs). (**A**) Transfection with the wild-type human LRRK2 (WT-hLRRK2) and mutant human G2019S LRRK2 (GS-hLRRK2) was confirmed via a comparison with the vector using RT-PCR. Glyceraldehyde 3-phosphate dehydrogenase (GAPDH) was used as the loading control. (**B**) Time-dependent cell viability was measured using the CCK-8 assay. Treatment with Triton X-100 was used as the control for 100% death, and untransfected samples were used as the control for 100% survival. Analytical significance between the vector and GS-hLRRK2 or WT-hLRRK2 and GS-hLRRK2 at each time point is indicated by four asterisks. *N* = 3 (average of duplicate assays); two-way analysis of variance (ANOVA) with Bonferroni’s post hoc analysis; ns, not significant; *, *p* < 0.05; ****, *p* < 0.0001.

Before confirming the molecular changes induced by the transfection of rASTROs with the cloned vectors (WT-hLRRK2 and GS-hLRRK2), the expression of the inserted DNA was validated (Figure 1A), along with the effect of the transfected DNA on rASTROs. Transfection with GS-hLRRK2 significantly decreased cell viability compared to that with vector and WT-hLRRK2 in a time-dependent manner (Figure 1B). However, after day 3, transfection with WT-hLRRK2 significantly decreased cell viability compared to that with the vector. Although the expression of WT-hLRRK2 promoted the normal function of LRRK2, which is advantageous for cellular function and maintenance, the increased LRRK2 kinase activity because transfection with WT-hLRRK2 would induce LRRK2 kinase-mediated cellular toxicity.

### 3.2. Alterations in Neurotrophic Factors and Proinflammatory Cytokines due to G2019S LRRK2 Expression in rASTROs

**Figure 2:**
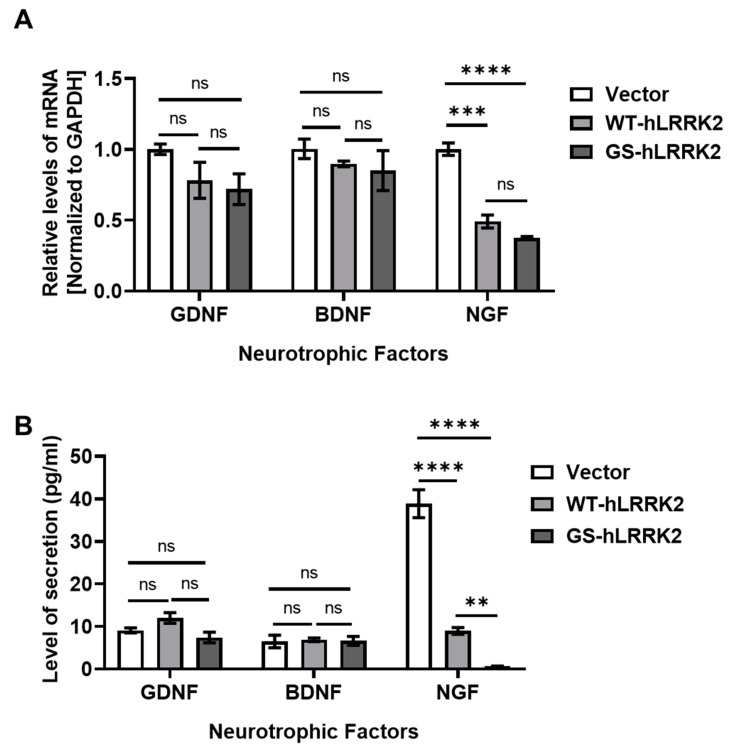
Levels of neurotrophic factors following ectopic expression of LRRK2 in rASTROs. To validate the changes in protective functions of rASTROs, the levels of neurotrophic factors in the culture media were determined via an mRNA quantification assay (**A**) and an enzyme-linked immunosorbent assay (ELISA) (**B**). These factors included glial cell line-derived neurotrophic factor (GDNF), brain-derived neurotrophic factor (BDNF), and nerve growth factor (NGF). *N* = 3 (average of duplicate assays); two-way ANOVA with Bonferroni’s post hoc analysis; ns, not significant; **, *p* < 0.01; ***, *p* < 0.001; ****, *p* < 0.0001.

To determine the effects of transfection with WT-hLRRK2 or GS-hLRRK2 on the expression of neurotrophic factors in rASTROs, we estimated the levels of four factors: brain-derived neurotrophic factor (BDNF), glial cell line-derived neurotrophic factor (GDNF), and nerve growth factor (NGF). The expression levels of GDNF and BDNF did not significantly change after transfection. However, transfection with WT-hLRRK2 and GS-hLRRK2 significantly reduced NGF expression in rASTROs (Figure 2A). Although transfection with GS-hLRRK2 caused a slight decrease in NGF expression compared to that with WT-hLRRK2, no significant difference was found. The levels of secreted neurotrophic factors were also estimated; however, no significant changes in GDNF and BDNF levels were found among the different groups. Notably, NGF levels significantly decreased in the GS-hLRRK2 group compared to those in the vector and WT-hLRRK2 groups. In addition, the levels of NGF were significantly decreased in the WT-hLRRK2 group compared to those in the vector control group (Figure 2B).

**Figure 3:**
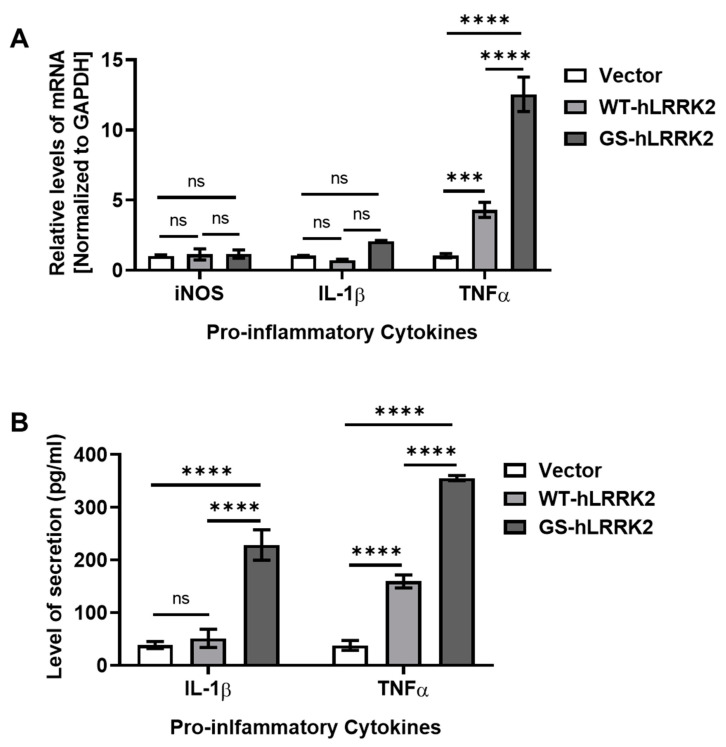
Levels of proinflammatory cytokines following ectopic expression of LRRK2 in rASTROs. To assess neuroinflammation, we examined the levels of proinflammatory cytokines, including inducible nitrogen oxide synthase (iNOS), interleukin-1 β (IL-1β), and tumor necrosis factor α (TNFα), in the culture media via an mRNA quantification assay (**A**) and ELISA (**B**). *N* = 3 (average of duplicate assays); one-way ANOVA with Bonferroni’s post hoc analysis; ns, not significant; ***, *p* < 0.001; ****, *p* < 0.0001.

To determine the effects of the inserted LRRK2 DNAs on neuroinflammation, we estimated the levels of proinflammatory cytokine, including inducible nitrogen oxide synthase (iNOS), interleukin-1 β (IL-1β), and tumor necrosis factor α (TNFα). Transfection with the DNAs did not change the levels of iNOS and IL-1β. GS-hLRRK2-expressing rASTROs exhibited the highest levels of TNFα; however, TNFα levels were also significantly increased in WT-hLRRK2-expressing rASTROs (Figure 3A). The secretion of IL-1β via ectopic expression of WT-hLRRK2 did not differ from that with the vector; however, ectopic expression of GS-hLRRK2 significantly increased the release of IL-1β compared to that induced by the vector or WT-hLRRK2. GS-hLRRK2 induced the highest level of TNFα secretion. Moreover, WT-hLRRK2 induced a significant increase in TNFα levels compared to that induced by the vector (Figure 3B). The remarkable increases in the levels of the proinflammatory cytokines, IL-1β and TNFα, and the decrease in NGF affected the cell viability of GS-hLRRK2-expressing rASTROs. Furthermore, the relatively mild increase in TNFα and decrease in NGF in cells transfected with WT-hLRRK2 compared to those transfected with GS-hLRRK2 may have affected the viability of cells transfected with WT-hLRRK2. Overall, the expression and extracellular secretion patterns of neurotrophic factors and inflammatory cytokines were similar between rASTROs transfected with WT-hLRRK2 and those transfected with GS-hLRRK2.

### 3.3. Astrogliosis in G2019S LRRK2-Expressing rASTROs

**Figure 4:**
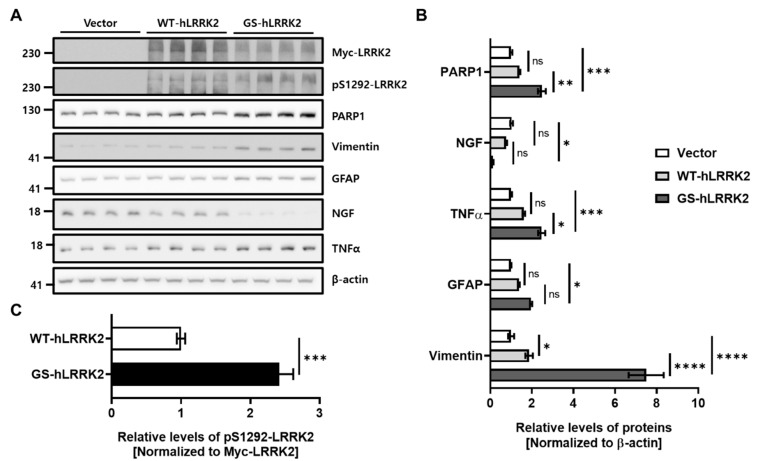
Alteration of astrogliosis due to the ectopic expression of LRRK2 in rASTROs. (**A**) Western blot analysis using the lysates of cells transfected with the indicated DNA. (**B**) Levels of glial fibrillary acidic protein (GFAP), vimentin, and poly(ADP-ribose) polymerase 1 (PARP1), TNFα, and NGF were measured and analyzed. β-Actin was used as the loading control for normalization. Transfection with WT-hLRRK2 and GS-hLRRK2 was confirmed using a Myc tag and auto-phosphorylation of LRRK2 on serine 1292 (pS1292-LRRK2). The level of pS1292-LRRK2 was estimated using Myc-LRRK2 (**C**). *N* = 4; one- or two-way ANOVA with Bonferroni’s post hoc analysis; ns, not significant; *, *p* < 0.05; **, *p* < 0.01; ***, *p* < 0.001; ****, *p* < 0.0001.

Western blot analysis was performed to verify the effect of GS-hLRRK2 expression on astrogliosis. The expression levels of WT-hLRRK2 and GS-hLRRK2 in rASTROs were also confirmed (Figure 4A). Markers of astrogliosis, including glial fibrillary acidic protein (GFAP) and vimentin, significantly increased in cells transfected with GS-hLRRK2 compared to those transfected with vector. In addition, transfection with WT-hLRRK2 resulted in a significant increase in vimentin levels and a non-significant increase in GFAP levels (Figure 4A,B) compared to transfection with the vector. As astrogliosis markers increased due to the expression of human LRRK2, the protein levels of TNFα and NGF, the two most markedly changed among the previously verified neuroinflammatory and neurotrophic factors, were assessed. The levels of TNFα and NGF were significantly increased by the ectopic expression of GS-hLRRK2 (Figure 4A,B). To validate the effect of increased astrogliosis, enhanced neuroinflammation, and reduced neurotrophic factor levels because of GS-hLRRK2 transfection on cell viability, the levels of PARP1 (Poly [ADP-ribose] polymerase 1), which is involved in apoptosis and TNFα-induced DNA damage, were analyzed. Notably, PARP1 levels increased with increases in GFAP, vimentin, and TNFα and a decrease in NGF (Figure 4A,B), thereby decreasing cell viability (Figure 1B). Although the ectopic expression of WT-hLRRK2 in rASTROs increased the kinase activity of LRRK2, that of GS-hLRRK2 was approximately 2.4-fold higher than the kinase activity of WT-hLRRK2 (Figure 4A,C). Collectively, our findings indicate the promotion of astrogliosis by the expression of WT-hLRRK2 or GS-hLRRK2; however, the remarkable increases in astrogliosis and TNFα and a decrease in NGF were only observed in cells transfected with GS-hLRRK2.

### 3.4. G2019S LRRK2-Expressing rASTROs Promote Neurotoxicity and the Dopamine Synthesis Pathway

**Figure 5:**
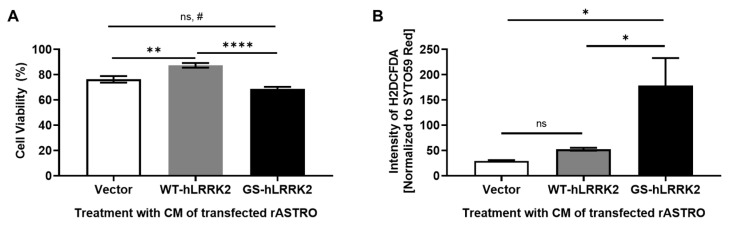
Viability and oxidative stress in N27 cells treated with the conditioned medium (CM) of transfected rASTROs. To determine the effects of astrocytes on dopaminergic neuronal health, N27 cells were treated with the CM of transfected rASTROs for 48 h; the CM was collected after 5 days of rASTRO culture. The viability of N27 cells following treatment with the CM of transfected rASTROs was determined using the CCK-8 assay (**A**), and the reactive oxygen species levels were detected using H2dCFDA (**B**). *N* = 5 (average of duplicate assays); one-way ANOVA with Tukey’s post hoc analysis; ns, not significant; *, *p* < 0.05; **, *p* < 0.01; ****, *p* < 0.0001; #, *p* < 0.05 based on Student’s *t*-test.

**Figure 6:**
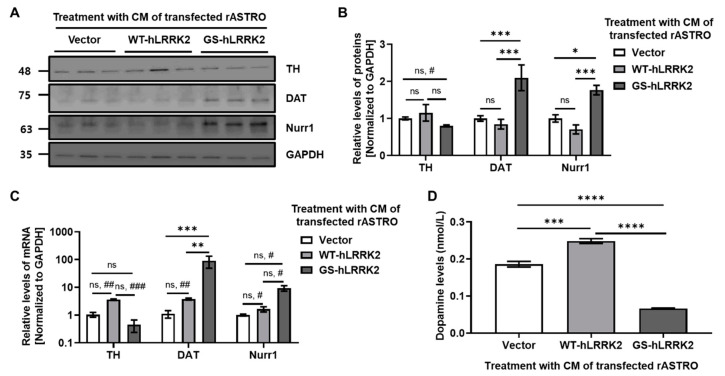
Changes in the dopaminergic synthesis pathway due to treatment with the CM of transfected rASTROs. (**A**,**B**) Levels of proteins involved in the dopamine synthesis pathway, including tyrosine hydroxylase (TH), dopamine transporter (DAT), and Nurr1. (**C**) mRNA expression of TH, DAT, and Nurr1 in N27 cells treated with the CM of transfected rASTROs. *N* = 3; two-way ANOVA with Bonferroni’s post hoc analysis; ns, not significant; *, *p* < 0.05; **, *p* < 0.01; ***, *p* < 0.001; #, *p* < 0.05 based on Student’s *t*-test; ##, *p* < 0.01 based on Student’s *t*-test; ###, *p* < 0.001 based on Student’s *t*-test. (**D**) Dopamine levels in the culture media of N27 cells treated with the CM of transfected rASTROs determined using ELISA. *N* = 4 (average of triplicate assays); one-way ANOVA with Bonferroni’s post hoc analysis; ***, *p* < 0.001; ****, *p* < 0.0001.

Finally, we investigated the overall implications of G2019S LRRK2 expression on dopamine neurons. Aliquots of CM containing transfected rASTROs were used to treat N27 cells for 48 h. Treatment with the CM of WT-LRRK2-transfected rASTROs significantly increased cell viability compared to treatment with that of vector-transfected rASTROs. In contrast, treatment with the CM of GS-hLRRK2-transfected rASTROs decreased cell viability compared to treatment with that of WT-hLRRK2- and vector-transfected rASTROs (Figure 5A). Furthermore, using the H2DCFDA reagent, the ROS levels in N27 cells were increased by treatment with the CM of GS-hLRRK2-transfected rASTROs compared to that with the CM of vector- or WT-hLRRK2-transfected rASTROs (Figure 5B). To identify issues associated with the functional aspect of dopamine neurons, we investigated the levels of tyrosine hydroxylase (TH, the rate-limiting enzyme of dopamine synthesis), dopamine transporters (DAT), and Nurr1 (which is involved in the transcription of TH and DAT). The mRNA and protein levels of TH were significantly decreased by treatment with the CM of GS-hLRRK2-transfected rASTROs compared to that with the CM of vector-transfected rASTROs. The levels of DAT and Nurr1 were significantly increased by treatment with the CM of GS-hLRRK2-transfected rASTROs compared to that with the CM of vector- or WT-hLRRK2-transfected rASTROs (Figure 6A–C). To validate the effect of treatment with the CM of transfected rASTROs on the dopamine synthesis pathway in N27 cells, we measured the levels of dopamine. Treatment with the CM of GS-hLRRK2-transfected rASTROs significantly decreased dopamine levels compared to that with the CM of vector- or WT-hLRRK2-transfected rASTROs (Figure 6D). Treatment with the CM of WT-hLRRK2-transfected rASTROs significantly increased the TH, DAT, and Nurr1 proteins and dopamine levels. This may be attributed to the mild stress induced by increased TNFα and decreased NGF in the CM of WT-hLRRK2-transfected rASTROs. A previous study found a decrease in TH levels and striatal dopamine in a double knock-out of the TNFα receptors (TNFR1 and TNFR2) compared to wild-type [19], verifying the protective effect of TNFα on dopaminergic neurons.

Collectively, our findings indicate that GS-hLRRK2-expressing rASTROs may be neurotoxic and may affect the regulation of the dopamine synthesis pathway in dopaminergic neurons.

## 4. Discussion

Astrocytes, colloquially known as star cells, are multifunctional brain cells that play critical roles in overall brain function and maintenance. Astrocytes are responsible for the formation of the physical structure of the brain, provide a framework for neurons, and organize and support their placement, which is crucial for proper brain function [1]. Notably, astrocytes play a key role in the composition and maintenance of the blood–brain barrier, a protective layer that separates the brain from the bloodstream [2]. Furthermore, astrocytes enhance communication between brain cells by influencing signal transmission between neurons and information processing in the brain and modulating signal strength [3]. Astrocytes also regulate the metabolic processes in the brain by providing nutrients to the neurons, aiding in waste removal, and maintaining the ionic balance in the extracellular space around neurons [4]. Astrocytes are also involved in the repair of nervous system damage following a traumatic injury. These cells can divide and occupy spaces formed due to neuronal injury, thereby actively contributing to the repair process and aiding in the formation of scar tissue [5]. Astrocytes possess high-affinity uptake systems for neurotransmitters, such as glutamate and gamma-aminobutyric acid. Therefore, astrocytes can regulate the levels of neurotransmitters in the extracellular space, thereby influencing neuronal signaling [6,7]. Notably, astrocytes are involved in paracrine signaling, which significantly influences cytokine production within the brain’s milieu. Paracrine signaling in astrocytes involves the release of signaling molecules that affect neighboring cells, including other astrocytes, neurons, and microglia. This process is crucial for the coordination of cellular responses to various physiological and pathological stimuli [20]. Astrocytes produce cytokines, which inflict autocrine or paracrine effects. In the context of paracrine signaling, astrocytes release cytokines, which affect the behavior of other cells and sometimes that of the releasing cell itself, which can modulate neuroinflammation [21], thereby orchestrating neuronal activity. Some cytokines can participate in the modulation of synaptic transmission, the regulation of cerebral blood flow, and the brain’s response to injury or disease [22]. In pathological conditions, such as neuroinflammation, the paracrine release of cytokines by astrocytes may get dysregulated, contributing to neurodegenerative disease progression [23].

In this study, we sought to understand the function of astrocytes in maintaining a healthy dopaminergic neuronal framework. Neurotrophic factors and neuroinflammation are associated with the maintenance of neuronal health [8,9]. Astrocytic NGF levels have been reported to regulate TH levels in dopaminergic neurons [24,25]. Several studies have also demonstrated the key roles of LRRK2 in neuroinflammation [13,17]. The release of proinflammatory cytokines during neuroinflammation may be neurotoxic and aggravate neurodegeneration [26]. Previous studies have revealed that GS-hLRRK2-expressing astrocytes fail to protect neurons due to impaired homeostasis of calcium ions [27] and mitochondrial malfunction [11]. Alterations in DAT levels are associated with the loss of dopaminergic neurons [28], and Nurr1 serves as an essential transcriptional initiator of the dopamine synthesis pathway, regulating TH and DAT [29]. Based on our findings, the reduction of NGF (Figure 2; Figure 4A, B) in astrocytes due to transfection with GS-hLRRK2 (Figure 2) could decrease the level of TH in dopaminergic neurons (Figure 6). Similarly, the GS-hLRRK2-associated induction of IL-1β and TNFα in astrocytes (Figure 3; Figure 4A, B) could increase neurotoxicity (Figure 5). Due to GS-hLRRK2 expression in astrocytes, the decrease in NGF and the increase in TNFα and IL-1β may propagate via paracrine or autocrine pathways, which can gradually increase neuroinflammation and exacerbate neuronal death, leading to PD progression. Notably, inflammation may be the mechanism underlying reduced dopamine synthesis and release [30].

The modification of DAT by ROS, including dopamine-derived quinones, can reduce dopamine uptake [31]. DAT elevation can aid dopamine recycling and compensate for the disrupted synthesis of the dopamine precursor l-3,4-dihydroxyphenylalanine by lowering TH levels. The modification of DAT by neuroinflammation-induced oxidative stress may drive Nurr1 expression to increase DAT expression for dopamine uptake. Moreover, reduced dopamine levels may promote Nurr1 expression to enhance the transcription of enzymes involved in the dopamine synthesis pathway (Figure 6). However, an increase in DAT due to these symptoms, rather than overcoming dopamine deficiency, may make dopaminergic neurons vulnerable via a vicious cycle and accelerate PD progression, as increased DAT expression results in the loss of dopaminergic neurons in mice [28]. Although the viability of N27 cells was altered by treatment with the CM of WT-hLRRK2- and GS-hLRRK2-transfected rASTROs, no severe loss of N27 cells was observed. Interestingly, individuals who harbor LRRK2 mutations have a relatively late onset of PD due to the upregulation of other neurotrophic factors that may play a protective role against the death of dopaminergic neurons. Future studies should further evaluate the effects of neurotrophic factors and neuroinflammatory cytokines on neuronal survival and dopamine synthesis using antagonists, inhibitors, and gene silencers in animal models.

The association between LRRK2 and α-synuclein may be critical for PD progression [32]. Previously, we found that α-synuclein accumulates in neurons with upregulated LRRK2 kinase activity, as observed with the G2019S LRRK2 mutant and following rotenone treatment [12]. Moreover, accumulated α-synuclein in neurons can be released into the extracellular space and then transmitted to other cells, including neurons [33], microglia [34], and astrocytes [35]. Our previous studies also revealed that neuron-derived α-synuclein provokes microglia activation and neuroinflammation [26]. Oligomeric α-synuclein can promote the inflammation of astrocytes [36]. In addition, inflammatory responses amplify the cell-to-cell propagation of α-synuclein [32,34,35]. The vicious cycle, orchestrated by the interplay between α-synuclein and LRRK2 across different brain cells, might lead to the accumulation of α-synuclein over decades and deteriorating neurodegeneration in patients with PD. Future studies should investigate the crosstalk between LRRK2 and α-synuclein, with a specific focus on the interaction between G2019S LRRK2 mutant-expressing neurons and microglia, as well as astrocytes. Additionally, the interaction between G2019S LRRK2 mutant-expressing astrocytes and microglia should also be explored.

LRRK2 has recently emerged as a potential therapeutic target for PD in academic and industrial research. Therefore, future studies are needed to determine whether LRRK2 kinase inhibitors can effectively mitigate the LRRK2-associated adverse effects in brain cells, including astrocytes, microglia, and dopaminergic neurons.

## 5. Conclusions

The role of G2019S LRRK2 in astrocytes might contribute to the degeneration of dopaminergic neurons via reduced NGF and induced pro-inflammatory cytokines. Further studies are required to confirm the effects of each of these molecular mechanisms on neurotoxicity and dopamine production pathways. Future studies should also explore the association between astrocytes and other brain cells, considering the synergistic actions of G2019S LRRK2 and α-synuclein. Finally, the effects of LRRK2 inhibitors on the crucial functions of astrocytes should be examined. This knowledge will be instrumental in developing strategies to alleviate PD progression.

## Figures and Tables

**Table 1:** Primer sequences.

Gene		Sequence (5′-3′)
Human *LRRK2*		CTTCTGAGATGAGTTTTTGTTCCTCGAC
Myc taq		GAGTCATGATGACAGCACAGC
*BDNF*	Forward	GTTCGGCATTGCGAGTTCCAG
	Reverse	TTGAGCACGTGATCGAAGAGC
*GDNF*	Forward	GACTCCAATATGCCCGAAGA
	Reverse	TAGCCCAAACCCAAGTCAGT
*NGF*	Forward	ACCTCTTCGGACACTCTGGA
	Reverse	GTCCGTGGCTGTGGTCTTAT
*TNFα*	Forward	ACTGAACTTCGGGGTGATTG
	Reverse	GCTTGGTGGTTTGCTACGAC
*TGFβ*	Forward	GCAACAACGCAATCTATGAC
	Reverse	CCTGTATTCCGTCTCCTT
*IL-1β*	Forward	CCAGGATGAGGACCCAAGCA
	Reverse	TCCCGACCATTGCTGTTTCC
*iNOS* (*NOS2*)	Forward	CACCTTGGAGTTCACCCAGT
	Reverse	ACCACTCGTACTTGGGATGC
*TH*	Forward	TTCCCCATGTTCAACGGACC
	Reverse	GCGAGCACAGTAATCACCTTC
*DAT*	Forward	GGAAGCTGGTCAGCCCCTGCT
	Reverse	GAATTGGCGCACCTCCCCTCTG
*NURR1*	Forward	CTACGCTTAGCATACAGGTC
	Reverse	TTCCTTGAGCCCGTGTCT
*GAPDH*	Forward	CAAGTTCAACGGCACAGTCAAG
	Reverse	ACATACTCAGCACCAGCATCAC

**Table 2:** Primary and secondary antibodies.

Antibody	Company	Catalog Number	Dilution
PARP (poly [ADP-ribose] polymerase)	Cell Signaling Technology, Beverly, MA, USA	9542S	1/1000
NGF	Santa Cruz Biotechnology, Dallas, TX, USA	Sc-518166	1/200
TNFα	Santa Cruz Biotechnology	sc-52746	1/200
β-actin	Santa Cruz Biotechnology	sc-47778	1/3000
Vimentin	Santa Cruz Biotechnology	sc-6260	1/5000
GFAP (gial fibrillary acidic protein)	Santa Cruz Biotechnology	sc-33673	1/5000
TH (tyrosine hydroxylase)	Cell Signaling Technology	2792S	1/1000
DAT (dopamine transporter)	Santa Cruz Biotechnology	sc-14002	1/1000
NURR1/NR4A2 (nuclear receptor-related 1/nuclear receptor 4A2)	Proteintech, Rosemont, IL, USA	10975-2-ap	1/1000
GAPDH (glyceraldehyde 3-phosphate dehydrogenase)	Santa Cruz Biotechnology	sc-32233	1/5000
Anti-LRRK2/Dardarin, NeuroMab clone N241A/34 Pure IgG	UC Davis/NIH NeuroMab Facility	75-253	1/100
Peroxidase-conjugated AffiniPure goat anti-mouse IgG (H+L)	Jackson Immunoresearch Laboratories Inc., West Grove, PA, USA	115-035-003	1/5000
Peroxidase-conjugated AffiniPure goat anti-rabbit IgG (H+L)	Jackson Immunoresearch Laboratories Inc.	111-035-144	1/5000

## Data Availability

The datasets generated and/or analyzed in the current study are available from the corresponding author upon reasonable request.
